# Green synthetic natural carbon dots derived from *Fuligo Plantae* with inhibitory effect against alcoholic gastric ulcer

**DOI:** 10.3389/fmolb.2023.1223621

**Published:** 2023-07-07

**Authors:** Yusheng Zhao, Guoliang Cheng, Yushan Gao, Luming Cui, Yafang Zhao, Yifan Zhang, Yu Tian, Yan Zhao, Yue Zhang, Huihua Qu, Hui Kong

**Affiliations:** ^1^ School of Traditional Chinese Medicine, Beijing University of Chinese Medicine, Beijing, China; ^2^ School of Life Sciences, Beijing University of Chinese Medicine, Beijing, China; ^3^ Centre of Scientific Experiment, Beijing University of Chinese Medicine, Beijing, China

**Keywords:** *Fuligo Plantae*, carbon dots, alcoholic gastric ulcer, hemostasis, antiinflammatory, antioxidant, intestinal flora

## Abstract

**Introduction:** *Fuligo Plantae* (FP), the ash that sticks to the bottom of pots or chimneys after weeds burn, has long been used for its hemostatic effects and treatment of gastrointestinal bleeding. Nevertheless, the active ingredient of FP still needs to be further explored.

**Methods:** The microstructure, optical and chemical properties of FP-CDs were characterized. An alcohol-induced gastric ulcer model was utilized to evaluate whether pre-administration of FP-CDs alleviated gastric bleeding symptoms and ameliorated gastric mucosal barrier disruption. In addition, the feces of each group of rats were extracted for 16S rDNA genome sequencing of intestinal flora.

**Results:** FP-CDs with a diameter ranging from 1.4–3.2 nm had abundant chemical groups, which may be beneficial to the exertion of inherent activity. FP-CDs alleviated alcohol-induced gastric ulcer, as demonstrated by activating the extrinsic coagulation pathway, alleviating inflammation, and suppressing oxidative stress levels. More interestingly, FP-CDs can improve the diversity and dysbiosis of intestinal flora in rats with alcohol-induced gastric ulcer.

**Conclusion:** These comes about illustrate the momentous inhibitory effects of FP-CDs on alcoholic gastric ulcer in rats, which give a modern methodology for investigating the effective ingredient of FP, and lay an experimental basis for the application of FP-CDs in the clinical treatment of alcoholic gastric ulcer.

## 1 Introduction

Alcoholic gastric ulcer (AGU) is a common intense gastrointestinal disease. Oral intake of a large amount of alcohol will directly damage the gastric mucosa epithelial cells and destroy the gastric mucosal barrier, thereby causing acute gastric mucosal congestion, erosion, and even superficial ulcers (Liu et al., 2021; Ren et al., 2021). With the change of lifestyle, the incidence of alcohol-related diseases is increasing year by year. Epidemiological surveys have found that the probability of gastrointestinal diseases in people who drink is much higher than those who do not drink (Sumbul et al., 2011). Alcohol is one of the direct factors of acute gastric ulcer, which makes the research on the avoidance and treatment of alcohol-induced gastric ulcer and its mechanism a hot spot in gastric health research.

AGU is a heterogeneous disease with high incidence and complex etiology. Commonly used clinical drugs include histamine receptor antagonists, cytoprotective agents, antibiotics, proton pump inhibitors and prostaglandins analogs, but these treatment methods will produce adverse effects side effects, and have the disadvantages of easy recurrence and long course of treatment (Rickard, 2016; Maes et al., 2017; Rahman et al., 2021). Therefore, there is an urgent need for a more secure and lower-cost medicate to treat AGU. In this regard, the research on the anti-gastric ulcer activity of nanoparticles in the field of nanomaterials has attracted many scholars and showed good therapeutic effects.

As a novel type of carbon-based nanomaterials, carbon dots (CDs) with unique advantages such as predominant photoluminescence (Zhao et al., 2021), good biocompatibility (Cui et al., 2021), tunable chemical properties (Lin et al., 2017), and excellent water dispersibility (Vinoth Kumar et al., 2021) have attracted widespread attention in many fields, including bioimaging (Ansari et al., 2021), drug delivery (Chen et al., 2021) and cancer therapy (Nocito et al., 2021). Due to their significant advantages, the biological activities of CDs in medicine, including anti-tumor (Zhang W. et al., 2021), bacteriostatic (Wang et al., 2020), anti-viral (Tong et al., 2020), anti-inflammatory (Hu et al., 2021), immune regulation (Zhang M. et al., 2021), hemostasis (Yan et al., 2017), analgesia (Zhang et al., 2020), have been confirmed by various scientific experiments, which opens entirely novel drugs for the discovery of effective remission or cure of certain clinical diseases.

*Fuligo Plantae* (FP), named Zao-xia-hui in Chinese, is the ash that weeds attach to the bottom of the pot or chimney after burning, and has a long-time medicinal history. It is the finished product by gently scraping the ash from the bottom of the pot or chimney and sifting out impurities. Ancient Chinese classic medical books documented that FP can be utilized to treat diverse symptoms, such as hemorrhage, inflammation, food accumulation and jaundice. After observing that FP has a wide range of civil applications, we have extensive interest in whether it has a certain effect and its unexplained underlying mechanism. Interestingly, it is found that the high-temperature carbonization strategy of FP is exceptionally comparable to the one-step pyrolysis strategy to get ready CDs. Recently, we discovered the CDs from aqueous extracts of FPs by modern instruments including transmission electron microscopy and optical instruments, and used a series of strategies to extract FP-CDs. Therefore, it is speculated that FP-CDs with electron exchange capacity and diverse functional groups may be the effective ingredients within the treatment of bleeding gastric ulcer disease.

Herein, an alcohol-induced gastric ulcer model was used to evaluate whether pre-administration of FP-CDs ameliorated gastric mucosal barrier disruption and alleviated gastric bleeding symptoms. The therapeutic effect of FP-CDs was assessed by observing macroscopic images and pathological changes of gastric tissue, measuring the levels of inflammatory indicators and oxidative stress indicators, and further exploring the impact on the intestinal flora.

## 2 Materials and methods

### 2.1 Materials

Alcohol and chloral hydrate were purchased from Beijing Solarbio Science & Technology Co., Ltd. (Beijing, China). Dulbecco’s Modified Eagle Medium (DMEM) and foetal bovine serum (FBS) were purchased from Corning Co., Ltd. (New York, United States). Cell counting kit-8 (CCK-8), tumour necrosis factor-α (TNF-α), interleukin (IL)-6 and IL-1β were brought from Beijing Bairuiji Biotechnology Co., Ltd. (Beijing, China). Alanine transaminase (ALT), aspartate aminotransferase (AST), Malondialdehyde (MDA), superoxide dismutase (SOD) and glutathione (GSH) kits were purchased from Nanjing Jiancheng Bioengineering Institute of China (Nanjing, China). The analytical grade chemicals and reagents were purchased from China National Pharmaceutical Industry Corporation Ltd. (Beijing, China). All the experiments were performed utilizing deionized water (DW).

### 2.2 Animals

Male grown-up Sprague–Dawley (SD) rats weighing 210.0 ± 10.0 g were purchased from Beijing Jinmuyang Co., Ltd. (Beijing, China) under the following rearing conditions: temperature (24.0 ± 1.0) °C, Relative humidity 55%–65%, 12 h light/dark cycle, free access to food and water.

### 2.3 Synthesis of FP-CDs

**FIGURE 1:**
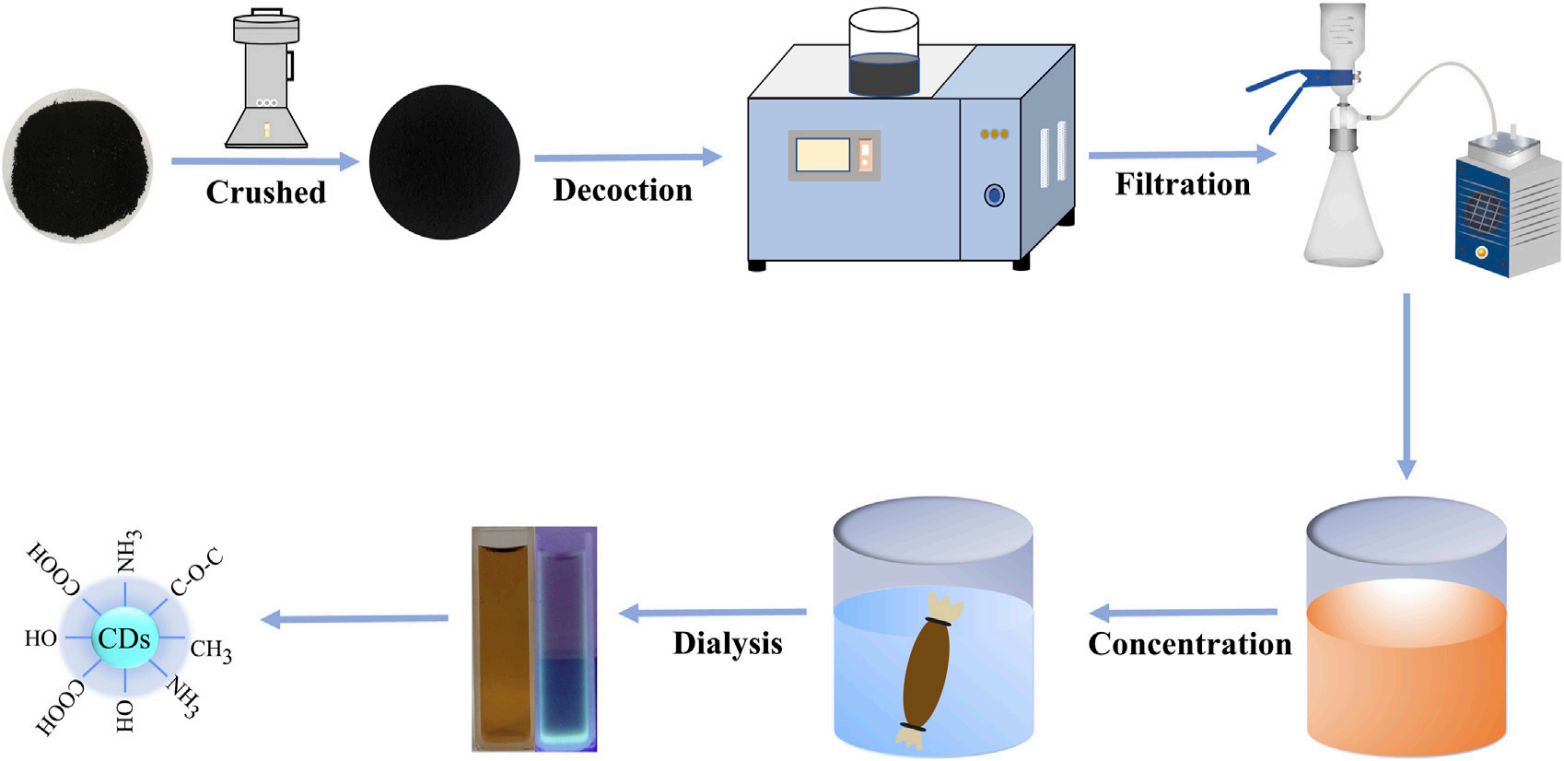
The flowchart for the preparation process of carbon dots derived from *Fuligo Plantae* (FP-CDs) by one-step calcination method.

To put it succinctly, 50 g FP was ground into fine powder and boiled 2 times with DW in a 100°C water bath for 1 h each time. Subsequently, the water decoction was filtered with 0.22 μm organic microporous membrane and concentrated to 50 mL. At that point, the concentrated was dialyzed against 1,000 Da molecular weight dialysis membrane in a measuring utensil filled with DW. Finally, the gotten FP-CDs were put away at 4°C until assist use. The schematic diagram of the experimental protocol for the preparation of FP-CDs is exhibited in Figure 1.

### 2.4 Characterization of FP-CDs

The morphology, distribution and microstructure information of FP-CDs were revealed by transmission electron microscopy (TEM) (Tecnai G220, FEI Company, United States), while the atomic lattice spacing of FP-CDs was uncovered utilizing a high-resolution TEM (JEN-1230, Japan Electron Optics Laboratory, Japan). The emission wavelength and electronic transition characteristics of FP-CDs were gotten by utilizing ultraviolet (UV-Vis) spectrophotometer (CECIL, Cambridge, United Kingdom) and fluorescence (FL) spectrophotometer (F-4500, Tokyo, Japan) respectively. The functional group properties of FP-CDs were further characterized with Fourier transform infrared (FTIR) spectroscopy (Thermo Fisher, Fremont; California, United States) and X-ray photoelectron spectroscopy (XPS) (ESCALAB 250Xi, Thermo Fisher Scientific, United States). The polydispersity index (PDI) value of FP-CDs was measured with dynamic light scattering (DLS).

### 2.5 Cytotoxicity assay of FP-CDs

In order to assess the hazards of FP-CDs and investigate its safety, the CCK-8 experiments were performed to recognize the cytotoxicity of FP-CDs to RAW 264.7 mouse macrophage, human gastric epithelial cell lines (GES-1) and human LO2 hepatocyte. The 3 cells were cultured in DMEM medium containing 20% fetal bovine serum in a humidified 5% CO_2_ atmosphere at 37°C. Subsequently, the 3 cells were seeded in a 96-well plate at a density of 1 × 10^5^ cells per 100 μL/well and incubated for 24 h, respectively. Then, different concentrations of FP-CDs (650, 325, 162.5, 81.25, 40.63, 20.31, 10.16 μg/mL) were added to the designated wells for 24 h and the control cells were treated with DMEM medium. After these plates were washed thrice with PBS, 10 μL of CCK-8 is added to the plate and incubated for 4 h. Additionally, a microplate reader (Biotek, Vermont, United States) was utilized to record the absorbance of each well. Finally, the cell viability (%) calculation formula is as follows:

$$Cell\;Viability\;\left(\%\;of\;control\right)=\left(Ae-Ab\right)/\left(Ac-Ab\right)\times 100$$ (1)

where Ae, Ab, and Ac represent the absorbance of the experimental, blank and control groups, respectively, at 450 nm.

### 2.6 Biosafety experiment in rats

Twenty rats were randomly divided into the following two groups (*n* = 10 in each) according to the administration situation: control group (normal saline, 0.5 mL, i.g.), administration intervention group (FP-CDs, 9.98 mg/kg, i.g.). Next, the reaction and mortality of each group of mice within 24 h were observed for 5 consecutive days. After 4 days of administration, rats in each group fasted for 24 h during which they drank freely. On the 5th day of administration, after 3 h of administration, rats were anesthetized by intraperitoneal injection of 4% chloral hydrate (0.40 g/kg). Blood samples were collected from rats via the abdominal aorta by blood taking needles and vacuum blood collection tubes (Becton Dickinson Medical Instrument Co., Ltd., Shanghai, China) to detect indexes of hepatic function (ALT and AST). Subsequently, the heart, liver, spleen, lung, kidney, and thymus of rats in each group were extracted in 4% tissue fixation solution, and the organs of each group were observed by HE staining.

### 2.7 Coagulation parameter measurements

Sixty rats were randomly divided into the following six groups (*n* = 10 in each) according to the administration situation: control group (normal saline, 0.5 mL, i.g.), model (normal saline, 0.5 mL, i.g.), positive group (Yunnan Baiyao powder, 250 mg/kg, i.g.), and FP-CDs at different doses [low (L): 2.49 mg/kg, medium (M): 4.99 mg/kg, high (H): 9.98 mg/kg; i.g.). After 4 days of administration, rats in each group fasted for 24 h during which they drank freely. On the 5th day of administration, after 2 h of administration, all groups were given to 95% alcohol (10 mL/kg, i.g.) except the control group, which acquired an equal amount of normal saline. After being modelled with alcohol for 1 h, rats were anesthetized by intraperitoneal injection of 4% chloral hydrate (0.40 g/kg). Next, Abdominal aortic blood was infused into prepacked 3.2% sodium citrate (blood: citrate: 1:9, v/v) centrifuge tubes and permitted to respond for at slightest 30 min. Subsequently, blood samples were centrifuged at 750 × g for 15 min to get supernatant. Activated partial thromboplastin time (APTT), thrombin time (TT), prothrombin time (PT), and fibrinogen content (FIB) were determined utilizing a programmed coagulation analyser.

### 2.8 Models of alcohol-induced acute gastric ulcer model in rats and drug treatment

The AGC model was established as previously reported (Gao et al., 2020). A total of 60 SD rats were randomly divided into the following six groups (*n* = 10 in each) according to the administration situation: control group (normal saline, 0.5 mL, i.g.), model (normal saline, 0.5 mL, i.g.), positive (ranitidine, 50 mg/kg, i.g.) and FP-CDs at different doses [low (L): 2.49 mg/kg, medium (M): 4.99 mg/kg, high (H): 9.98 mg/kg; i.g.]. After 4 days of administration, rats in each group fasted for 24 h during which they drank freely. On the 5th day of administration, after 2 h of administration, all groups were given to 95% alcohol (10 mL/kg, i.g.) except the control group, which gotten an equal volume of normal saline.

### 2.9 The calculation of gastric ulcer area and gastric ulcer inhibition rate

After being modelled with alcohol for 1 h, rats were euthanized. Subsequently, the abdominal cavity was opened to separate the gastric tissue, cut along the more noteworthy curvature, and after that washed with ice-cold saline until there was no content or bloodshot. The stomach was stretched on a clean ice box with the mucous membrane facing up, and was instantly captured by a computerized camera to watch the ulcer zone. The ratio of mucosal ulcer area in each group was automatically assessed by ImageJ software (v1.8.0v, MD, United States). The ulcer index (UI) and percentage inhibition (Xin et al., 2021) were calculated by the following formulas:

$$UI\;\left(\%\right)=zone\;of\;gastric\;ulcer/area\;of\;entirety\;stomach\times 100$$ (2)

Percentage Inhibition (%) = (UI in model group-UI in each group)/UI in model group × 100. (3)

### 2.10 Histopathological evaluation

After the gastric tissue was watched and captured, portion of the gastric tissue was quickly put in 4% paraformaldehyde, got dried out, embedded in paraffin, and recoloured with HE. The recoloured segments were shot and watched beneath a magnifying lens at amplifications of ×100 and 200 ×. Subsequently, the pathological changes of gastric mucosa in each group were used for comparative observation.

### 2.11 Examinations of biochemical indicators

The remaining portion gastric tissues of rats were weighed and processed with 9 times volume of pre-cooling PBS solution to make 10% gastric tissue homogenate. Next, the supernatant centrifuged at 750 × g for 15 min was utilized to detect the contents of inflammatory cytokines (TNF-α, IL-6, and IL-1β) and the contents of oxidative stress indicators (MDA, SOD, and GSH) utilizing corresponding kits according to the instructions.

### 2.12 16S rDNA genome sequencing for intestinal flora

New feces of each group of rats were collected with sterile forceps and put away at −80°C for gene sequencing. Total DNA in feces was extracted according to the instructions of the DNA extraction kit (MN NucleoSpin 96 Soi, US), and the V3-V4 region of 16S rDNA was opened with primers with barcodes. The primer sequences were 338F: ACT​CCT​ACG​GGA​GGC​AGC​A, 806R: GGACTACHVGGGTWTCTAAT. The PCR amplification products were at that point recuperated by gel cutting and measured concurring to electrophoresis. Afterwards, the purified amplification products were blended in rise to sums, ligated with sequencing connectors, and sequenced using Illumina Novaseq 6,000 (Beijing Biomarker Cloud Technology Co., Ltd., China).

### 2.13 Statistical analysis

All data from experiment were carried out utilizing IBM SPASS (version 25.0, Chicago, IL). The values were represented as means ± standard deviation. Multiple comparisons were performed employing a one-way analysis of variance (ANOVA) followed by the least-significant difference (LSD) test. *p* < 0.05 and *p* < 0.01 were considered statistically significant.

## 3 Result

### 3.1 Characterization of FP-CDs

**FIGURE 2:**
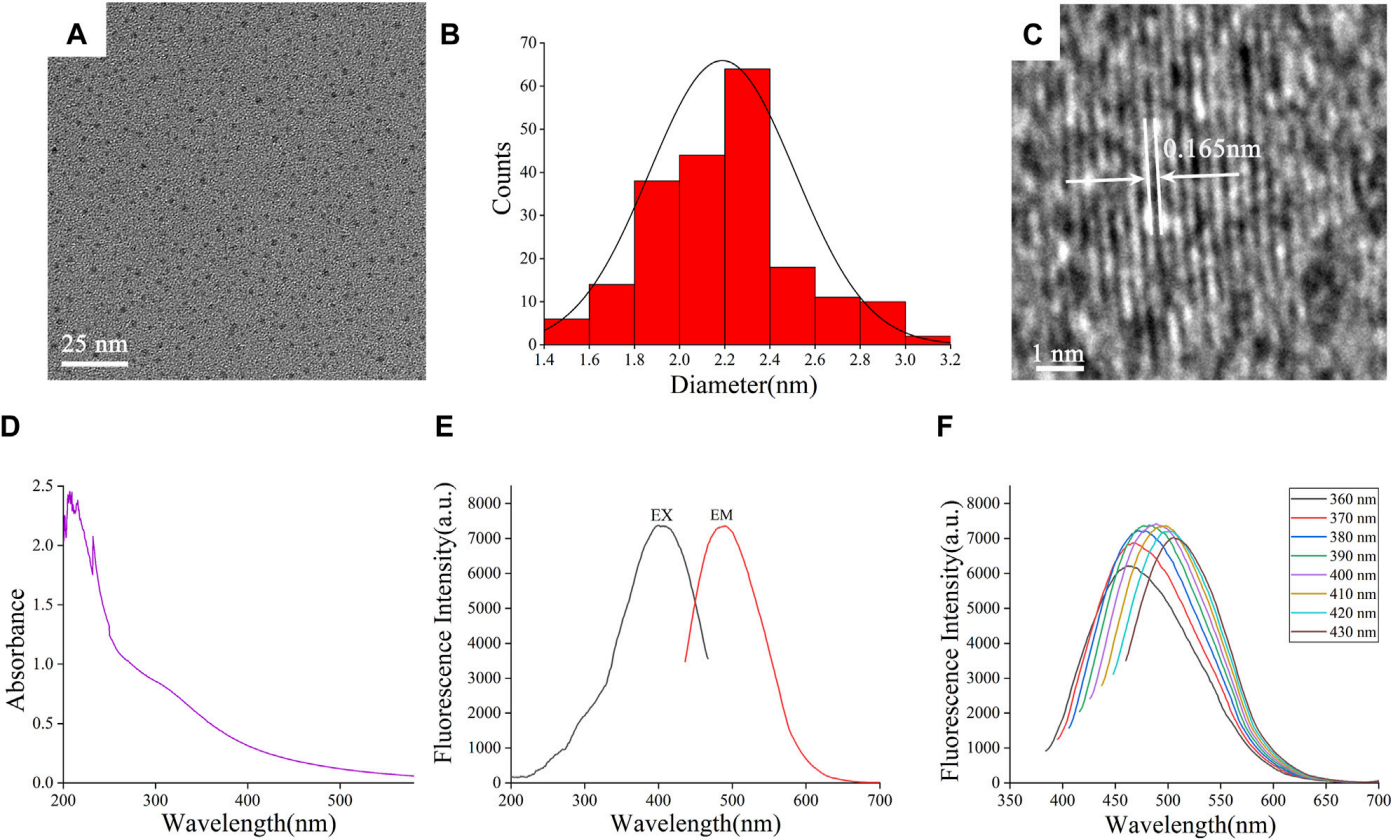
Morphological and optical characterizations of FP-CDs. **(A)** Transmission electron microscopy (TEM) images of FP-CDs displaying ultra-small particles. **(B)** Particle size distribution histogram of FP-CDs. **(C)** High-resolution TEM image of FP-CDs and lattice spacing of ASAC-CDs (in the middle). **(D)** Ultraviolet–visible spectrum. **(E)** Fluorescence spectra. **(F)** Fluorescence spectra of FP-CDs with different excitation wavelengths.

The TEM image (Figure 2A) directly exhibited that the FP-CDs were spherical-like particles consistently distributed within the field of vision without self-evident conglomeration. The particle sizes of FP-CDs were fundamentally scattered between 1.4 and 3.2 nm (Figure 2B), which acclimated to the normal distribution characteristics and was obtained by statistical analysis of more than 200 particles with the support of ImageJ software (Bhamore et al., 2019). As illustrated in Figure 2C, the high-resolution TEM image appeared well-resolved lattice fringes and a lattice spacing of 0.165 nm, which was near to the value of the crystal plane of graphite. These morphological findings are consistent and dependable with past survey reports (Wang et al., 2021).

The optical properties of FP-CDs were assist examined utilizing UV-Vis spectrophotometer and FL spectrophotometer. The UV-Vis spectra of FP-CDs (Figure 2D) displayed several distinct absorption peaks between 200 and 240 nm, which may be caused by the π-π* electronic transition of the conjugated C=C bonds and aromatic sp^2^ domains (Sun et al., 2021). At the same time, the absorption peak around 300 nm may be caused by the n-π* electronic transition caused by the unsaturated heteroatom bond. As shown in Figure 2E, it can be seen from the FL spectra of FP-CDs that the maximum emission wavelength (EM_max_) is at 490 nm when excited at 399 nm. Further analysis of the fluorescence characteristics (Figure 2F) can be found that as the excitation wavelength increases from 360 nm to 430 nm, the EM_max_ begins to red-shift and its fluorescence intensity showed up a drift of growing to start with and after that diminishing. According to literature reports (Wei et al., 2019), the optical properties of CDs vary with size, which may lead to changes in the density of CDs and the properties of sp^2^ sites. The phenomenon of the fluorescence wavelength red-shift is explained that it may be caused by the different emission traps on the surface of CDs of different sizes and the energy gap decreases with the increase of size under the condition of quantum confinement effect.

**FIGURE 3:**
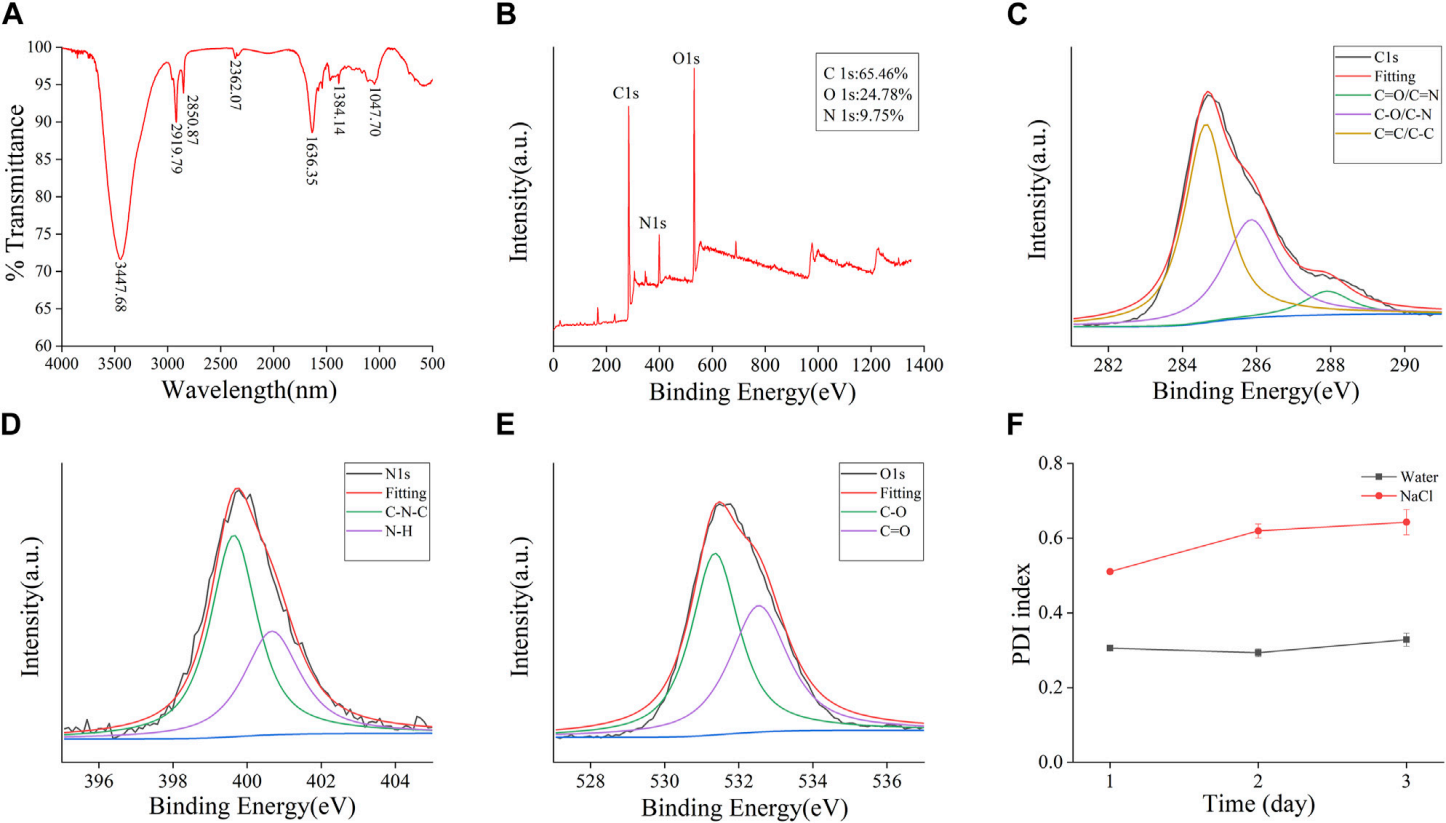
Functional groups and dynamic light scattering (DLS) analysis of the prepared FP-CDs. **(A)** Fourier transform infrared spectroscopy spectrum (FT-IR). **(B)** X-ray photoelectron spectroscopy survey (XPS), **(C)** C 1 s, **(D)** O 1 s, **(E)** N 1 s high-resolution survey spectrum and **(F)** The colloid stability of FP-CDs dispersed in water and NaCl measured by DLS.

The plenteous surface chemical groups and element composition of FP-CDs were advance characterized and analysed by FTIR and XPS techniques. Within the FTIR spectra (Figure 3A), the intense peak at 3,447 cm^−1^ was credited to the stretching vibration of O-H and N-H bonds. The absorption peaks at 2,919 cm^−1^ indicated the existence of -CH bonds, which was related to the binding of methyl or methylene groups to aliphatic hydrocarbons in FP-CDs. The peak at 1,636 cm^−1^ belonged to the stretching vibration peak of -C=O bonds, while the weakly absorbed peak at 1,384 cm^−1^ was associated with -C-N bonds (Manchala et al., 2021). Moreover, the peak was observed at 1,047 cm^−1^ means the existence of C-O-C bonds (Muhammad et al., 2019). Therefore, these results demonstrate that FP-CDs with abundant hydroxyl, amino and carboxyl groups on the surface have better water solubility and hydrophilicity.

Furthermore, three apparent element peaks in XPS spectra (Figure 3B) were observed clearly at 284.81, 399.84 and 531.67 eV, indicating that the FP-CDs were basically made up of C (65.46%), N (9.75%), and O (24.78%). Three obvious peaks at 284.63, 285.85, and 287.91 eV in the C 1 s (Figure 3C) spectrum were attributed to the presence of C=C/C-C, C-O/C-N and C=O/C-N, respectively. The N 1 s spectrum (Figure 3D) was divided into two apparent peaks at 399.64 and 400.67 eV, which were consistent with C-N-C and N-H bonds. Moreover, the O 1 s spectrum (Figure 3F) was isolated into two evident peaks at 531.36 and 532.54 eV, which confirmed the presence of C-O and C=O bonds (Radnia et al., 2020; Luo et al., 2021). The XPS experimental results are basically consistent with the FTIR characterization, indicating that FP-CDs contain various groups including carboxyl, hydroxyl, and amino groups, which can be credited to the multiphoton dynamic forms in the diverse oxygen-containing functional groups. In addition, the dispersion stability of FP-CDs is usually measured by DLS method. The colloidal stability of FP-CDs in water and NaCl at 25°C for 3 days was studied by DLS. The results (Figure 3F) showed that the PDI of FP-CDs in water did not change significantly.

### 3.2 Biosafety evaluation

**FIGURE 4:**
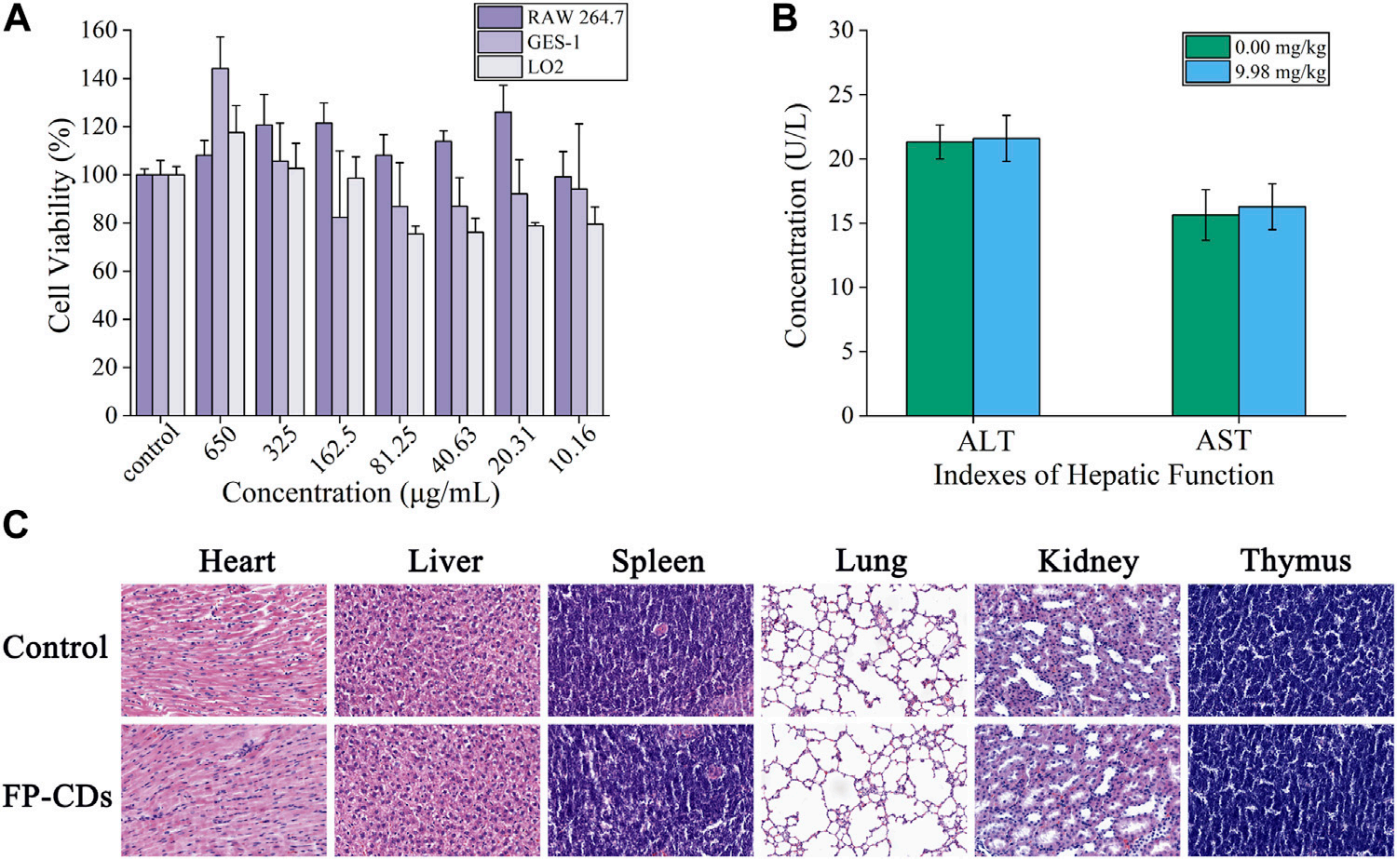
Biosafety experiment of FP-CDs. **(A)** Effect of different concentrations of FP-CDs on the viability of RAW264.7, GES-1 and LO2 cells via CCK-8 assay for 24 h. **(B)** Effects of FP-CDs (9.98 mg/kg) on indexes of hepatic function (ALT and AST) in normal rats. **(C)** Effect of FP-CDs (9.98 mg/kg) on pathological changes of the heart, liver, spleen, lung, kidney, and thymus in normal rats.

As potential unused nano-drug possessed a series of exceptional properties, CDs have been widely concerned about the security of clinical application. As shown in Figure 4A, FP-CDs with concentrations ranging from 10.16 to 650 μg/mL had little impact on the viability of RAW264.7, GES-1 and LO2 cells, and the overall survival rate was above 75%. In addition, FP-CDs even exhibited a certain promoting effect on cell viability at concentrations extending from 325 to 650 μg/mL. Animal serum biochemical parameters are one of the important indicators for evaluating organ function. As shown in Figure 4B, FP-CDs within the experimental dose range had no significant effect on indexes of hepatic function (ALT and AST) after continuous administration for 5 days, and there was no statistical difference between the groups compared with the normal group. Figure 4C showed that after 5 days of continuous intervention with FP-CDs, the structure of the heart, liver, spleen, lung, kidney, pancreas, and thymus of normal rats did not change significantly, which further suggests that FP-CDs have high security. The detection results of the biosafety experiment illustrated that FP-CDs have low toxicity, which has a certain potential reference value for the clinical use of nano-drugs in the future.

### 3.3 Effect of FP-CDs on the coagulation system

**FIGURE 5:**
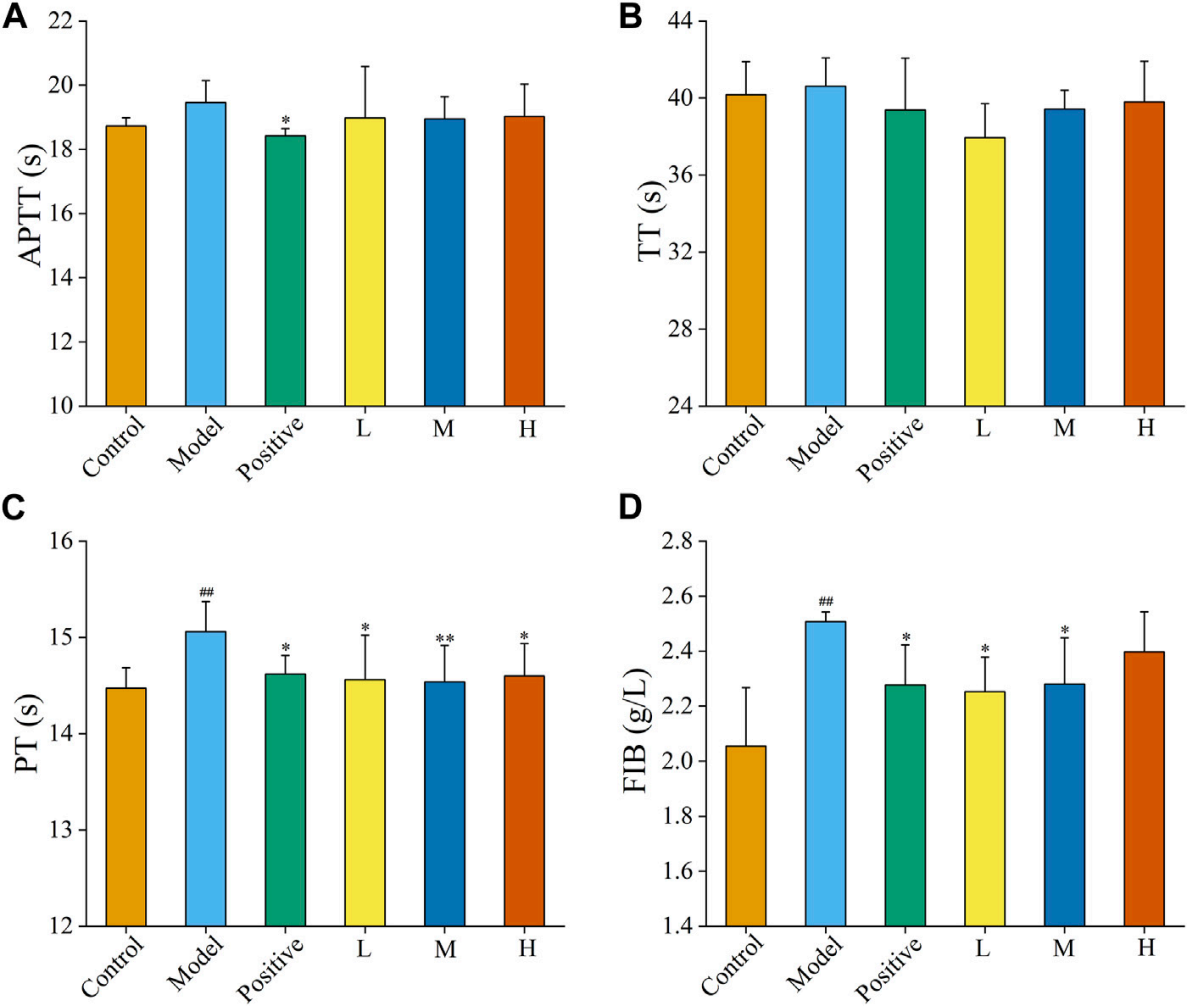
Effects on coagulation parameters. **(A)** activated partial thromboplastin time (APTT), **(B)** thrombin time (TT), **(C)** prothrombin time (PT), and **(D)** fibrinogen (FIB). Analysis of rats treated with control group, model group, positive (Yunnan Baiyao) group, or low (L), medium (M), or high (H) doses of FP-CDs (2.49, 4.99, and 9.98 mg/kg, respectively). Significantly different compared with the control group at ^##^ *p* < 0.01, significantly different compared to the model group at **p < 0.01 and *p < 0.05.

The haemostatic mechanism of FP-CDs evaluated by assessed four coagulation parameters (APTT, TT, PT, and FIB). As illustrated in Figures 5A, B, APTT and TT values were not essentially distinctive among the four treatment groups in comparison with the model group. Figures 5C, D exhibited that the values of PT and FIB in the model group were elevated (*p* < 0.01) in comparison with the control group. In addition, the PT and FIB values of Yunnan Baiyao group and FP-CDS group at low, medium, and high doses were alleviated in contrast with the model group (*p* < 0.05), which indicated that the haemostatic efficacy of FP-CDs may be related to the exogenous coagulation route.

### 3.4 FP-CDs ameliorated alcohol-induced structural abnormalities

**FIGURE 6:**
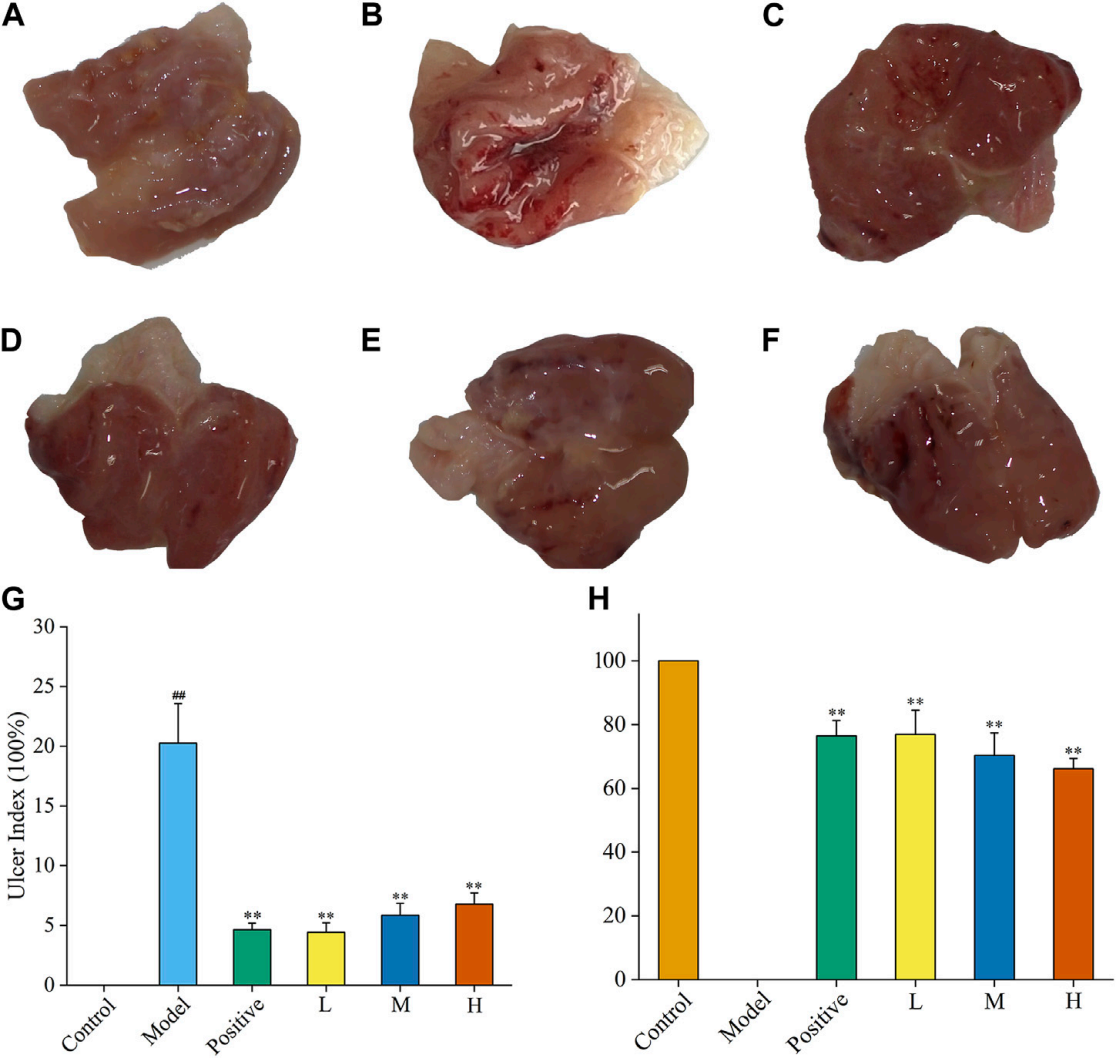
Effects of FP-CDs on the gastric mucosa in rats with alcohol-induced ulcers. **(A)** control group, **(B)** model group, **(C)**positive (ranitidine) group, **(D)** low (L), **(E)** medium (M), and **(F)** high (H) doses of FP-CDs (2.49, 4.99, and 9.98 mg/kg, respectively), **(G)** the ulcer index, **(H)** percentage inhibition of each group. Significantly different compared with the control group at ^##^ *p* < 0.01, significantly different compared to the model group at **p < 0.01.

As a gastric mucosal attack factor, excessive high concentration of alcohol can directly damage gastric mucosal tissue, stimulate gastrointestinal motility, and cause mucosal oedema, haemorrhage necrosis, and inflammation. In this experiment, the inhibitory efficacy of FP-CDs on AGU in rats caused by excessive oral alcohol was investigated by evaluating the morphological changes in the stomach of rats in each group, calculating the ratio of ulcer index and inhibition rate. Compared with the control group (Figure 6A), gastric tissue (Figure 6B) after oral alcohol administration exhibited marked spasms and extensive dark red streaks, demonstrating serious haemorrhage of the gastric mucosa. Moreover, in contrast to the model group, the gastric mucosal bleeding of rats treated with ranitidine (Figure 6C) and FP-CDs (Figures 6D–F) was significantly reduced, and only a few punctate haemorrhages were seen, suggesting that FP-CDs alleviated the damage of gastric tissue to varying degrees.

Moreover, the UI and inhibition rate were calculated by ImageJ computer program to quantify the change of the lesion area and assess the gastroprotective efficacy of FP-CDs. As illustrated in Figures 6G, H, the UI of the rats in the model group accounted for a higher proportion in comparison with the control group, indicating that the gastric mucosal injury caused by alcohol was obvious (*p* < 0.01). In sharp contrast, the pre-administration group treated with ranitidine and FP-CDs obviously alleviated the ulcer area and exhibited higher inhibition rate (*p* < 0.01), in which the low-dose FP-CDS group with an inhibition rate of more than 76% had a stronger inhibition effect on ulcer injury.

**FIGURE 7:**
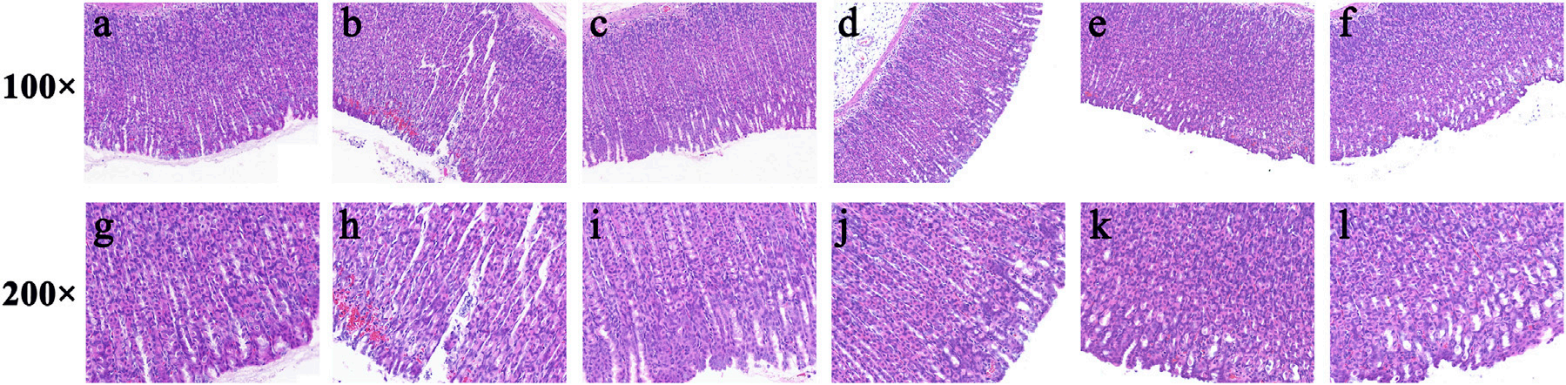
Effects of FP-CDs on histopathological changes in alcohol-induced gastric histopathological damage (magnification = × 100 and 200 ×). Histopathological sections of gastric tissue were stained with H&E. **(A,G)** control group, **(B,H)** model group, **(C,I)** positive (ranitidine) group, **(D,J)** low-dose FP-CDs group (2.49 mg/kg), **(E,K)** medium-dose FP-CDs group (4.99 mg/kg), and **(F,L)** high-dose FP-CDs group (9.98 mg/kg).

Furthermore, haematoxylin and eosin (HE) staining showed that the gastric mucosa in the control group (Figures 7A, G) was flat and smooth, and the cells were arranged in an orderly manner. While necrosis of mucosal cells, increased inflammatory cells and severe intervillous haemorrhage were clearly observed in the model group (Figures 7B, H). In contrast, oral administration of ranitidine (Figures 7C, I) and different doses of FP-CDs (L: Figures 7D, J, M: Figures 7E, K, H: Figures 7F, L) during pre-treatment significantly alleviated haemorrhage, oedema, and inflammatory cell infiltration, which further suggested the inhibitory effect of FP-CDs on alcohol-induced injury.

### 3.5 FP-CDs inhibited alcohol-induced inflammatory response

**FIGURE 8:**
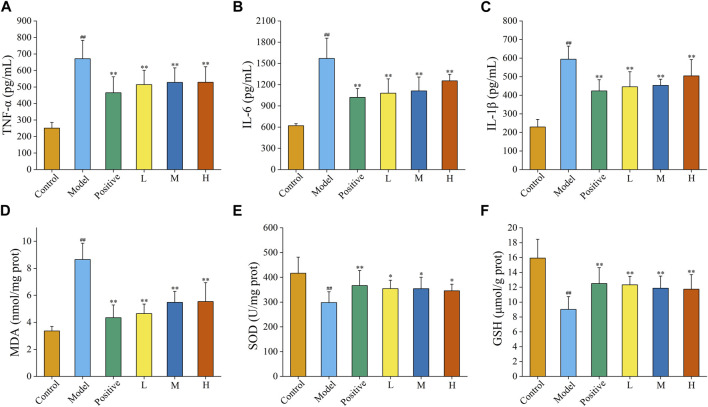
Effects of FP-CDs on inflammatory cytokines and oxidative stress in gastric tissue homogenate supernatant. **(A)** tumour necrosis factor-α (TNF-α), **(B)** interleukin (IL)-6, **(C)** IL-1β, **(D)** malondialdehyde (MDA), **(E)** superoxide dismutase (SOD), and **(F)** glutathione (GSH). Analysis of rats treated with control group, model group, positive (ranitidine) group, or low (L), medium (M), or high (H) doses of FP-CDs (2.49, 4.99, and 9.98 mg/kg, respectively). Significantly different compared with the control group at ^##^ *p* < 0.01, significantly different compared to the model group at **p < 0.01 and *p < 0.05.

Inflammation is one of the imperative underlying mechanisms of excess alcohol-induced AGU. As illustrated in Figure 8A, the contents of TNF-α within the model group (671.46 ± 111.51 pg/mL) was obviously upregulated (*p* < 0.01) in comparison to that of the control group (250.90 ± 34.69 pg/mL), suggesting that the model was successful (*p* < 0.01). Compared with that in the model group, the levels of TNF-α in the ranitidine (465.27 ± 96.79 pg/mL), low-dose (514.53 ± 86.66 pg/mL), medium-dose (527.82 ± 88.17 pg/mL) and high-dose (528.87 ± 93.51 pg/mL) groups produced a conspicuous reduction (*p* < 0.01).

Moreover, the content of IL-6 (Figure 8B) was obviously raised by alcohol in the model group (1,570.09 ± 287.61 pg/mL) compared to control group (620.17 ± 28.58 pg/mL), while the pre-administration of ranitidine (1,020.20 ± 124.22 pg/mL) and FP-CDs (L: 1,079.01 ± 201.86 pg/mL, M: 1,111.70 ± 195.81 pg/mL, H: 1,253.67 ± 91.96 pg/mL) could downregulate the index to varying degrees (*p* < 0.01).

Additionally, the level of IL-1β (Figure 8C) in the model group (594.18 ± 70.36 pg/mL, *p* < 0.01) also ascended in comparison to those in the control group (229.31 ± 40.83 pg/mL). In comparison to the model group, the levels of IL-1β in the ranitidine (423.21 ± 60.45 pg/mL), low-dose (445.49 ± 81.13 pg/mL), medium-dose (453.18 ± 32.92 pg/mL) and high-dose (504.20 ± 87.55 pg/mL) groups significantly decreased (*p* < 0.01).

### 3.6 FP-CDs attenuated alcohol-induced oxidative stress

Oxygen radicals in the body are associated with most causative factors for ulcers. Enhanced lipid peroxidase action and increased production of oxygen free radicals can trigger a decrease in cellular fluidity and peroxidation of membrane-structured unsaturated fatty acids, which can lead to cellular damage and further mucosal damage. In this study, changes in oxidative stress indicators were assessed by measuring the amount of MDA, SOD, and GSH in the gastric tissue (Figures 8D–F).

After being disturbed by alcohol, the level of MDA in the model group (8.66 ± 1.20 nmol/mg port, *p* < 0.01) was significantly higher than that in the control group (3.37 ± 0.33 nmol/mg port, *p* < 0.01). Meanwhile, the levels of SOD and GSH in the model group (SOD: 298.24 ± 43.28 U/mg prot, GSH: 9.02 ± 1.72 μmol/g prot) was significantly descending in comparison to that of the control group (SOD: 416.59 ± 64.84 U/mg prot, GSH: 15.93 ± 2.53 μmol/g prot, *p* < 0.01), which indicated that the modelling was successful.

Conversely, the contents of MDA in the positive group (4.35 ± 0.94 nmol/mg port) and FP-CDs groups (L: 4.65 ± 0.70 nmol/mg port, M: 5.48 ± 0.81 nmol/mg port, H: 5.54 ± 1.40 nmol/mg port) were significantly relieved in comparison to the model group (*p* < 0.01). In addition, pre-treatment with ranitidine (SOD: 366.66 ± 61.01 U/mg prot, GSH: 12.50 ± 2.12 μmol/g prot, *p* < 0.01) and low- (SOD: 354.53 ± 33.81 U/mg prot, *p* < 0.01; GSH: 12.33 ± 1.15 μmol/g prot, *p* < 0.05), medium-(SOD: 354.20 ± 46.24 U/mg prot, *p* < 0.01; GSH: 11.88 ± 1.62 μmol/g prot, *p* < 0.05), and high-doses (SOD: 345.81 ± 26.76 U/mg prot, *p* < 0.01; GSH: 11.73 ± 1.96 μmol/g prot, *p* < 0.05) of FP-CDs generated a noteworthy increment within the levels of Grass and GSH.

### 3.7 Effects of FP-CDs on intestinal microflora and metabolism

The composition and abundance ratio of intestinal flora have a close relationship with the health status of the body, and maintain a dynamic balance in the continuous development and change. In the state of alcohol stimulation, various homeostatic balances of the body are disrupted, resulting in intestinal flora imbalance, which in turn leads to the occurrence of startling gastrointestinal diseases. Here, we further investigated the impact of the more effective low-dose FP-CDs group on the intestinal flora.

**FIGURE 9:**
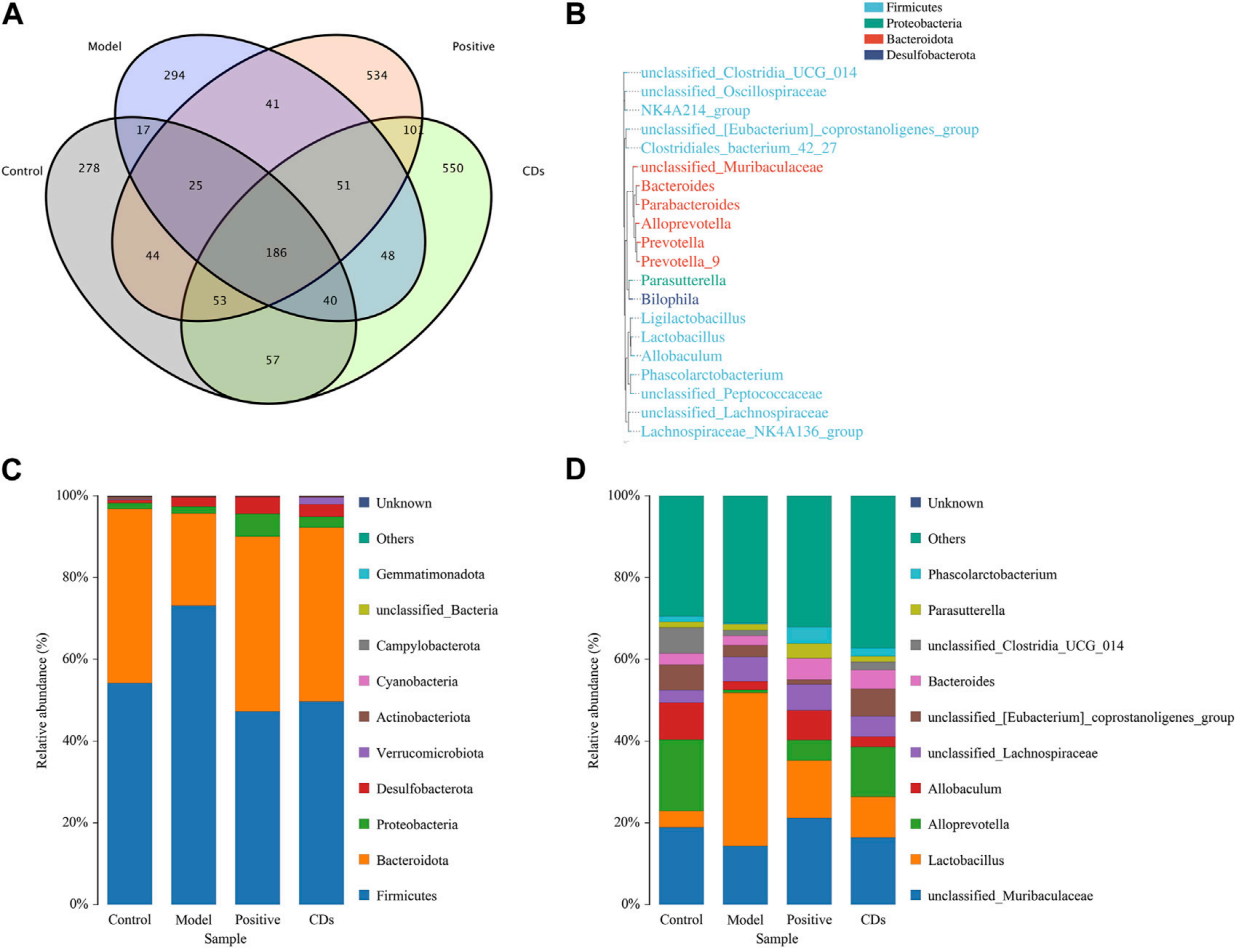
Effects of FP-CDs on the overall changes of intestinal flora in rats with alcohol-induced ulcers. **(A)** Venn diagram of intestinal flora of rats in each group, **(B)** schematic diagram of the phylogenetic tree at genus level for each group, **(C)** histogram of species distribution at phylum level for each group, **(D)** histogram of species distribution at genus level for each group.

The Venn diagram (Figure 9A) exhibited that a total of 186 species of intestinal flora in each group were common, apart from which, the other common flora number in control group and model group was 268. In contrast, that of common flora in the positive and FP-CDs groups was 308 and 336, respectively. In addition, the phylogenetic tree of the features at the genus taxonomic level (Figure 9B) displayed that the same colour genus name represented the same phylum, and was mainly divided into *Firmicutes*, *Bacteroidota*, *Proteobacteria*, and *Desulfobacterota*. The histograms of species richness at the phylum level (Figure 9C) and genus level (Figure 9D) were clearly observed the species composition and proportion of each group of samples, which further reflected the changes of species among each group of samples. The ranitidine and FP-CDs groups could significantly adjust the levels of *Firmicutes* (ranitidine: 25.89%, FP-CDs: 23.48%) and *Bacteroidota* (ranitidine: 20.30%, FP-CDs: 20.07%) in comparison to that of the model group at the phylum level. According to the intestinal flora structure analysis at the genus level, compared with the model group, the positive group and FP-CDs group could call back the proportions of *Alloprevotella* (ranitidine: 4.26%, FP-CDs: 11.49%) and *Bacteroides* (ranitidine: 3.00%, FP-CDs: 2.31%). At the same time, the proportions of *Lactobacillus* (27.45%) and *Phascolarctobacterium* (1.66%) makes the composition of the intestinal flora in FP-CDs group close to the level of the control group in comparison to that of the model group. These results implied that FP-CDs reversed the diversity and dysbiosis of the intestinal flora in rats with AGU, and bring it closer to the content of the control group.

## 4 Discussion

CDs, as a new type of nanomaterials with unique advantages, has shown great application potential in biomedical fields such as biological imaging and tumour therapy (Ansari et al., 2021; Zhang W. et al., 2021). At present, A lot of research focus on the exploration of disease treatment and the expansion of application fields, while ignoring the problem of raw materials. Researchers are turning their consideration to green precursors with therapeutic properties after considering the need for low toxicity and avoiding transport of goods (Zhao et al., 2020). Compared with chemically derived CDs, Chinese herbal medicine-derived CDs have the advantages of abundant raw material sources, simple preparation methods, good biocompatibility, good water solubility, low toxicity, and low cost, and are an ideal CDs precursor material selection (LuMaHuang et al., 2021). More notably, Chinese herbal medicine is rich in a variety of active ingredients that make them a direct route to heteroatoms. Therefore, in recent years, the research on the biological activity of CDs derived from Chinese herbal medicine has become the focus of many scholars at home and abroad.

Charcoal drugs are a kind of characteristic Chinese medicines with extensive pharmacological effects formed after similar high temperature carbonization of Chinese medicines from different sources. However, its material basis is still controversial. High temperature carbonization is a critical procedure in the processing of charcoal drugs, which is like the bottom-up pyrolysis of CDs. Different process parameters change the chemical bond splitting mode of the compounds in the raw medicinal materials, which in turn leads to changes in the particle size, crystal structure and biological activity of the formed CDs. In the preliminary work, our team demonstrated that CDs is the active substance basis of charcoal drugs. We have confirmed that CDs derived from *Pollen Typhae Carbonisata* (Yan et al., 2017) and *Selaginella tamariscina Carbonisata* (Zhang et al., 2019) can stimulate endogenous and exogenous coagulation pathways respectively to play a haemostatic role. In addition, CDs derived from different charcoal drugs showed similar efficacy, such as *Radix Sophorae Flavescentis*

*Carbonisata*-based CDs (Hu et al., 2021) and *Atractylodes macrocephala Carbonisata*-based CDs (Kong et al., 2021) both showed a certain anti-ulcer effect. The same charcoal-derived CDs also have various pharmacological activities, such as CDs derived from *Phellodendri Chinensis Cortex Carbonisata*, which have haemostatic effects, reduce kidney damage caused by snake venom, and treat psoriasis (AkhtarMalikArshad et al., 2021; Zhang M. et al., 2021). Moreover, CDs derived from *Artemisiae Argyi Folium Carbonisata* perform a certain selective antibacterial effect as well as anti-frostbite effect (Wang et al., 2020; Zhou et al., 2021).

Based on the revelation of previous studies, we used FP as the only biomass precursors for the first time to extract and isolate FP-CDs. XPS and FTIR spectra were utilized to demonstrate the plentiful hydrophilic groups on the surface of FP-CDs, corresponding to good water solubility and diffusion in solution. CCK-8 experiments showed that FP-CDs showed extremely low toxicity to RAW 264.7, GES-1 and LO2 cells. The data of the four coagulation tests illustrated that the PT and FIB values of the FP-CDs group were decreased, which indicated that the haemostatic effect of FP-CDs was mainly related to the activation of the extrinsic coagulation pathway or the fibrinogen system. On this basis, the biological activity of FP-CDs was further explored.

Gastric ulcers are a common and frequent digestive tract illness. Although emotional and food factors will directly affect the cure of gastric ulcer, heavy drinking is still one of the main factors for the recurrence of the disease (Yang et al., 2021). Excessive consumption of alcohol not only causes direct stimulation to the gastric mucosa, but also increases the mucosal lipid peroxidation and cell damage induced by free radicals formed in the process of alcohol digestion system. Alcohol can cause damage to the submucosal blood vessels in the stomach, which can dilate the blood vessels and change the blood flow, causing small blood vessels to rupture and bleed (Sistani Karampour et al., 2019). In addition, alcohol can increase gastric acid secretion, gastrointestinal motility disorders, abnormal changes in intestinal flora, and aggravate oxidative stress and inflammatory responses (Kong et al., 2021). Meanwhile, free radicals are also generated during the alcohol metabolism. Once these free radicals cannot be effectively removed, it will cause antioxidant damage to major biological molecules such as lipids, proteins, and DNA, and finally lead to alcoholic gastric damage (Aziz et al., 2019).

Inflammation plays a key role in alcoholic gastric ulcer. Excessive alcohol intake will activate the microcirculation blood vessels on the gastric mucosa to produce inflammatory cytokines, and destroy the normal functioning of gastric tissue (Arab et al., 2019). As one of the indicators for evaluating apoptosis, TNF-α can stimulate the generation of other inflammatory cytokines, further lead to the activation of neutrophils and the production of acute phase reactive protein, which in turn influences the blood oxygen supply of gastric mucosa (Lian et al., 2020). IL-1β and IL-6 are also critical pro-inflammatory factors in the body, which can induce neutrophil aggregation, thereby aggravating the inflammatory response (Lin et al., 2021). The data of this research showed that the high, medium, and low dose groups of FP-CDs could reduce the levels of TNF-α, IL-6 and IL-1β in the gastric tissue of model rats to different degrees to alleviate the acute inflammatory response caused by alcohol, which may be one of the potential gastroprotective mechanisms of FP-CDs.

Oxidative stress refers to the nonstop generation of reactive oxygen species (ROS) that over-burdens the capacity of the natural antioxidant resistance system, resulting in DNA, protein, and lipid damage, and is one of the critical reasons for the occurrence of AGU (Grigor’eva, 2020). After alcohol acts on the body, it can increase the production of ROS, resulting in a maladjustment between the production of oxygen free radicals and the antioxidant potential in the body, which in turn induces the aggregation of inflammatory cells and aggravates stomach damage (Xue et al., 2019). As an oxidation product, the content of MDA increases with the destruction of lipid film structure and function. SOD and GSH are important antioxidant enzymes in the human body, which decrease the production of free radicals and improve the body’s antioxidant capacity under excessive oxidative stress levels. From the experimental results, the high, medium, and low dose groups of FP-CDs can reduce the MDA content in the gastric tissue of model rats on the one hand, and at the same time ascend the levels of SOD and GSH, which indicates that FP-CDs can effectively inhibit alcohol-induced oxidative damage and improve the capacity to resist oxidation and purge free radicals.

The current commonly utilized anti-ulcer therapy may induce changes in the flora of each segment of the host’s gastrointestinal tract and further lead to the occurrence of other gastrointestinal illness. Therefore, the development of new drugs without negative effects on intestinal homeostasis is urgently needed. Here, we evaluated the efficacy of FP-CDs on rat intestinal microflora. The composition structure and differences of intestinal flora in rats were analysed from the phylum level. In this experiment, *Firmicutes* and *Bacteroidetes* were the dominant phyla in each group, and the ratio of *Firmicutes* to *Bacteroidetes* (F/B) was an important index reflecting the disorder of intestinal flora (Mousa et al., 2019). The F/B value of the model group increased, which was reversed after FP-CDs intervention. Analysis of the composition and differences of rat intestinal flora at the subordinate level showed that FP-CDs could restore the proportion of beneficial bacteria such as *Alloprevotella* and *Lactobacillus*. In conclusion, FP-CDs can improve the diversity and dysbiosis of intestinal flora in rats with AGU.

## 5 Conclusion

In summary, under the premise of using FP as the sole precursor, novel fluorescent FP-CDs with characteristic biological activity and less harmfulness were extracted and isolated, and proved to be effective active ingredients against gastric ulcer. The inhibitory effect of FP-CDs on gastric ulcer may be related to the mitigation of the levels of inflammatory factors and oxidative stress. In addition, FP-CDs can inhibit the symptoms of alcoholic gastric ulcer by modulating the structure of intestinal flora. This study not only provides a novel tactics for investigating the effective material basis of FP, but also lays an exploratory foundation for the application of FP-CDs in the clinical treatment of alcoholic gastric ulcer.

## Data Availability

The raw data supporting the conclusions of this article will be made available by the authors, without undue reservation.
